# Supplementary material for: ESR and Radiocarbon Dating of Gut Strings from Early Plucked Instruments
Source: Methods Protoc. 2020 Jan 28;3(1):13. doi: 10.3390/mps3010013 (PMC7189657; doi:10.3390/mps3010013)
Supplement: Supplementary file 1 [file mps-03-00013-s001.pdf]

Letter

ESR and Radiocarbon Dating of Gut Strings from Early Plucked Instruments

Sumiko Tsukamoto [1,*], Taro Takeuchi [2], Atsushi Tani [3], Yosuke Miyairi [4] and Yusuke Yokoyama [4]

[1] Leibniz Institute for Applied Geophysics, Stilleweg 2, Hannover 30655, Germany
[2] The consortium for Guitar Research, Sidney Sussex College, Cambridge CB2 3HU, UK; guitarouk@aol.com
[3] Graduate School of Human Development and Environment, Kobe University, Kobe 657-8501, Japan; tani@carp.kobe-u.ac.jp
[4] Atmosphere and Ocean Research Institute, The University of Tokyo, Chiba 277-8564, Japan; miyairi@aori.u-tokyo.ac.jp (Y.M.); yokoyama@aori.u-tokyo.ac.jp (Y.Y.)
* Correspondence: sumiko.tsukamoto@leibniz-liag.de

Received: 14 October 2019; Accepted: 17 January 2020; Published: date

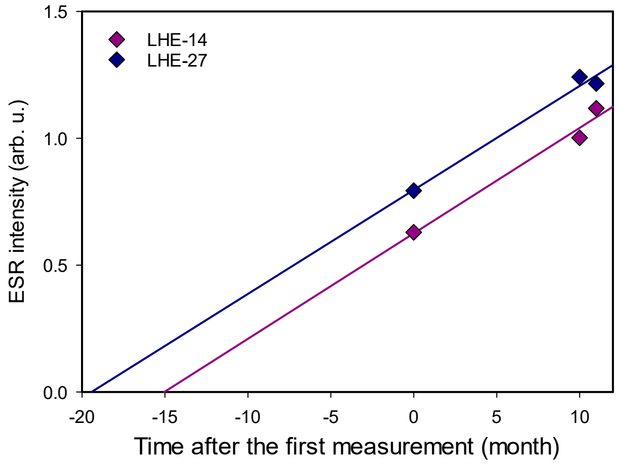

Figure S1. The increase of the Fe(III) (g = 2) signal after the first ESR measurement, April 2015 with time. The production time before the first measurement was roughly estimated by linearly extrapolating the fitted line to zero intensity. Data were normalised to the same factor as was used for Figure 3. Unfortunately further measurements were not achieved due to a problem with the ESR spectrometer.

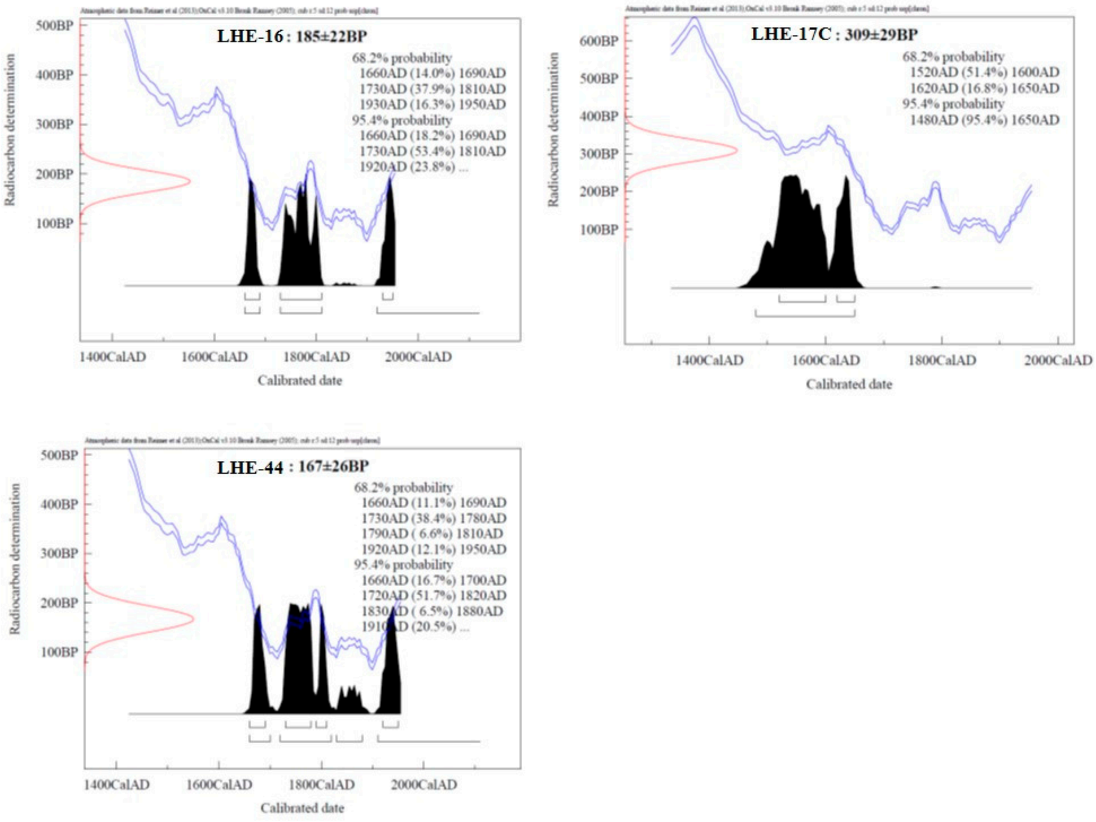

Figure S2. Results of calibration for the radiocarbon ages.

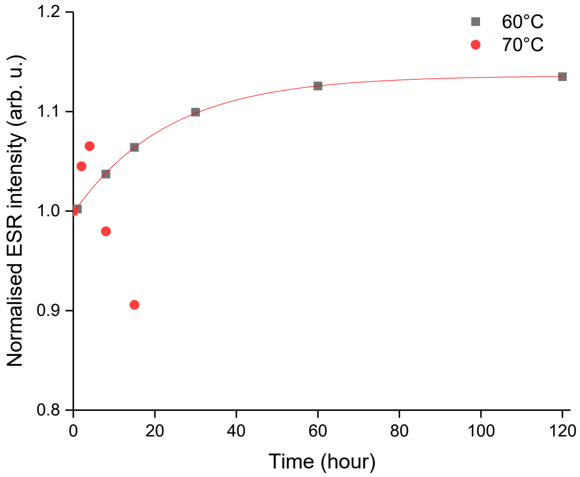

Figure S3. Result of the aging experiment of a string sample LHE-27 heated at 60°C and 70°C. The intensity of Fe(III) at g = 2 after different durations of heating is shown.
